# Sociodemographic and socioeconomic predictors of asymptomatic malaria among children and adolescents in rural Guinea: a longitudinal study

**DOI:** 10.3389/fpubh.2026.1891980

**Published:** 2026-07-23

**Authors:** Almamy Amara Toure, Sidikiba Sidibe, Soledad Colombe, Aboubacar Sidiki Magassouba, Abdoul Habib Beavogui, Abdoulaye Fodé Toure, Tiany Sidibe, Alexandre Delamou, Seni Kouanda

**Affiliations:** 1 Institut Africain de Santé Publique (IASP/USTA) of the University Saint Thomas D'Aquin, Ouagadougou, Burkina Faso; 2 National Institute of Public Health, Coyah, Guinea; 3 Department of Public Health, Faculty of Health Sciences and Techniques, Gamal Abdel Nasser University, Conakry, Guinea; 4 Centre d'Excellence Africain pour la Prévention et le Contrôle des Maladies Transmissibles (CEA-PCMT), Gamal Abdel Nasser University, Conakry, Guinea; 5 Department of Public Health, Institute of Tropical Medicine, Antwerp, Belgium; 6 Centre National de Formation et de Recherche en Santé Rurale de Mafèrinyah, Forécariah, Guinea; 7 Department of Public Health, Centre for Research in Reproductive Health, Conakry, Guinea

**Keywords:** adolescents, asymptomatic malaria, children, rural Guinea, socio-demographic and socio-economic predictors

## Abstract

**Introduction:**

Despite substantial progress in malaria control, asymptomatic malaria infection remains an important challenge for malaria elimination efforts in sub-Saharan Africa, particularly among older children and adolescents who may sustain ongoing transmissions. However, longitudinal evidence on the sociodemographic and socioeconomic determinants of asymptomatic malaria infection in rural areas remains limited. This study investigated the predictors and seasonal dynamics of asymptomatic malaria infection (AMI) in children and adolescents in Rural Guinea.

**Methods:**

We conducted a longitudinal cohort study in the rural sub-prefecture of Maferinya, Guinea, between March 2022 and February 2023. Children and adolescents aged 1–19 years were followed through repeated monthly visits covering both dry and rainy transmission seasons. Sociodemographic, socioeconomic, and parasitological data were collected using standardized questionnaires and microscopy. Mixed-effects logistic regression models with random intercepts for participants were used to identify the predictors of AMI while accounting for repeated measurements. Interaction analyses explored heterogeneity across age groups and seasons, and adjusted predicted probabilities were estimated using the final model.

**Results:**

Of 300 consented participants, 297 initiated study participation. After excluding 32 participants lost to follow-up (29 children, 0 adolescents), 268 participants (159 children aged 1–9 years and 109 adolescents aged 10–19 years) completed all nine scheduled monthly visits, contributing 2,412 visit-level observations to the final analysis. The predicted probabilities of malaria infection varied across age groups and seasons, with the highest probability observed among adolescents aged 10–14 years during the rainy season (5.2%, 95% CI: 1.3–18.6) and among those aged 15–19 years during the dry season (4.2%, 95% CI: 1.0–15.5). After adjustment, participants from households headed by individuals aged 35–49 years had higher odds of malaria infection than those from households headed by younger adults aged 22–34 years (aOR: 2.39, 95% CI: 1.04–5.48).

**Conclusion:**

Malaria infection among children and adolescents in rural Guinea is shaped by both seasonal transmission dynamics and household social characteristics. Older children and adolescents may represent important reservoirs of persistent transmission, highlighting the need for age-sensitive malaria surveillance and prevention strategies that extend beyond children aged under five years.

## 1 Introduction

Malaria remains a major global public health concern, with an estimated 282 million cases and 610,000 deaths reported worldwide in 2024 (1). Despite substantial progress over the past two decades, the global malaria burden has continued to rise in recent years, particularly in sub-Saharan Africa, which accounted for approximately 94% of malaria-related deaths in 2024 (1). Guinea ranks among the most heavily malaria-affected countries in the world, with an incidence of 315 cases per 1,000 people (2). The burden falls disproportionately on rural populations, where children are five times more likely to be infected than their urban counterparts (3).

Malaria transmission in rural settings is complex, particularly among school-aged children and adolescents. The prevalence of malaria among adolescents remains high, with notable disparities between rural and urban settings (4, 5). Emerging evidence from sub-Saharan Africa indicates that adolescents carry a substantial burden of infection and may contribute significantly to transmission, often through asymptomatic parasitaemia (6–9). This hidden burden is compounded by shifts in transmission dynamics; declining transmission in formerly high-endemic settings may reduce early life exposure and delay the acquisition of protective immunity, thereby increasing susceptibility to clinical malaria among school-age children and adolescents (10). Beyond their role in transmission, adolescents incur direct clinical costs, including heightened risks during pregnancy, where adolescent girls experience higher rates of peripheral and placental malaria than adult women (11), and broader impacts on development and educational attainment (10). Despite this growing recognition, malaria research and control efforts have historically focused on children under 5 years of age (12, 13) and pregnant women (14), neglecting the adolescent population.

Understanding why malaria remains so deeply entrenched in settings such as rural Guinea requires moving beyond purely biological or ecological explanations and situating the disease within a socio-structural framework. The World Health Organization Commission on Social Determinants of Health established that the conditions in which people are born, grow, live, work, and age, encompassing income, education, housing, food security, and access to essential services, are among the primary determinants of health inequities worldwide (15). Applied to malaria, this framework has generated a robust and growing body of evidence that socioeconomic position (SEP) and sociodemographic characteristics are fundamental structural drivers of infection risk (16, 17).

Importantly, biological and social factors do not operate in isolation; they interact and mutually reinforce one another. While biological susceptibility encompassing acquired immunity, nutritional status, host genetic traits, and co-infections undeniably shape individual vulnerability to malaria (18), this susceptibility is itself socially patterned. Younger children are biologically more vulnerable to severe malaria due to an immature immune system, whereas school-age children and adolescents, having acquired partial immunity through repeated exposure, more commonly harbor asymptomatic parasitaemia that perpetuates transmission (19). In addition, the intensity of cumulative exposure, quality of nutrition that modulates immune responses, and availability of treatment that prevents progression to severe disease are all determined, in large part, by household socioeconomic resources and the structural conditions of the living environment (20, 21). Such household socioeconomic resources include housing quality, food security, educational level, insecticide-treated net (ITN) use, and nutritional status (20).

Guinea is one of the least developed countries in the world, ranking 182nd out of 191 on the Human Development Index with a score of 0.46, placing it in the low human development category (22). An estimated 66.2% of the population is multidimensionally poor, with a mean deprivation intensity of 56.4%, reflecting simultaneous deficits in health, education, and living standards (23). First, the country's population is predominantly young, with over 51% under 18 years of age, and adolescents aged 10–18 years constitute approximately 23% of the national population, underscoring the public health relevance of this age group (22). In rural areas, where approximately 61.3% of the population resides (24), structural vulnerability to malaria is multidimensional and mutually reinforcing. Poverty in rural areas is more than double that in urban areas (55.4 vs. 22.4%) (22). This may restrict the ability to purchase, replace, or repair preventive materials such as ITNs and limit the financial capacity to seek care promptly when infection occurs. Despite the introduction of free malaria care services in 2010, direct and indirect costs associated with the malaria care pathways direct and indirect costs associated with the malaria care pathway remain a significant financial deterrent to timely care-seeking (25). Second, access to healthcare in rural areas of Guinea is severely constrained. Guinea's health facilities suffer from a critical lack of appropriate and properly maintained equipment, stable electricity, and hygienic working conditions, with 51% of the country's 1,383 public health facilities failing to meet the Ministry of Health's minimum physical structure standards, undermining the quality of services at the point of care (26). The poor state of rural roads further compounds access barriers, making it difficult for populations to reach health facilities, even when transportation is available (26). Third, housing in rural Guinea is predominantly traditional in construction, characterized by earthen walls and rudimentary roofing (3), which substantially increase indoor exposure to *Anopheles* mosquitoes during nocturnal biting hours. Evidence from multi-country analyses across 21 sub-Saharan African countries has demonstrated that children in traditional housing face significantly higher odds of malaria infection than those in modern and improved structures (27). In Guinea, this structural trap is compounded by profound gender inequalities; the country ranked 182nd out of 191 on the Gender Inequality Index in 2021 (22) which limits women's and girls' access to education, income, and decision-making power over health-seeking behavior, further amplifying malaria vulnerability. Therefore, examining socioeconomic and sociodemographic determinants in this specific context is not simply an extension of the existing literature to a new geography; it is essential because Guinea's particular constellation of structural vulnerabilities may generate mechanisms and risk gradients that are distinct from those documented in more heavily studied settings (16, 17).

Although the sociodemographic and socioeconomic determinants of malaria infection have been widely studied (13, 28, 29), several gaps remain. First, most available evidence is cross-sectional, limiting our understanding of how these predictors change and influence malaria risk. Second, rural populations are often treated as homogeneous despite substantial intra-rural variation in housing quality (30, 31), access to health services (32), environmental conditions (33), and socioeconomic status (33). Third, evidence remains limited for children and adolescents in rural Guinea, where local transmission patterns and household-level vulnerability may differ from those in other endemic settings.

This longitudinal study aimed to address these gaps by examining how sociodemographic and socioeconomic factors influence malaria transmission among children and adolescents in rural Guinea, explicitly accounting for intra-rural heterogeneity. By offering a more nuanced understanding of these relationships, our findings aim to inform the development of targeted and context-specific malaria control strategies for rural populations.

## 2 Methods

### 2.1 Study setting

The Republic of Guinea is located in West Africa and is bordered by Guinea-Bissau to the northwest, Senegal and Mali to the north, Ivory Coast to the east, Sierra Leone and Liberia to the south, and the Atlantic Ocean to the west. The country covers an area of approximately 245,857 km^2^. According to preliminary results from the Fourth General Population and Housing Census (RGPH-4), Guinea had an estimated population of approximately 17.7 million in 2025, corresponding to a national population density of approximately 71 inhabitants/km^2^, although the population distribution remains highly heterogeneous across the regions (24). Formerly known as “*The Rivers of the South*”, Guinea is one of the most water-abundant countries in West Africa. In addition to the region's longest coastline, it is the origin of 113 rivers that flow into neighboring countries, including the Niger River. To date, 1,161 watercourses have been recorded. Administratively, Guinea is divided into eight regions: Conakry (the capital), Kindia, Boké, Mamou, Labé, Faranah, Kankan, and N'Zérékoré (34). Mafèrinyah is a sub-prefecture within the Forécariah prefecture in the Kindia region. It is located approximately 60 km from Conakry, the national capital. The climate in Mafèrinyah is tropical and humid, with a distinct rainy season from May to October. This region is also known for its lush vegetation and rich natural environment.

**Figure 1:**
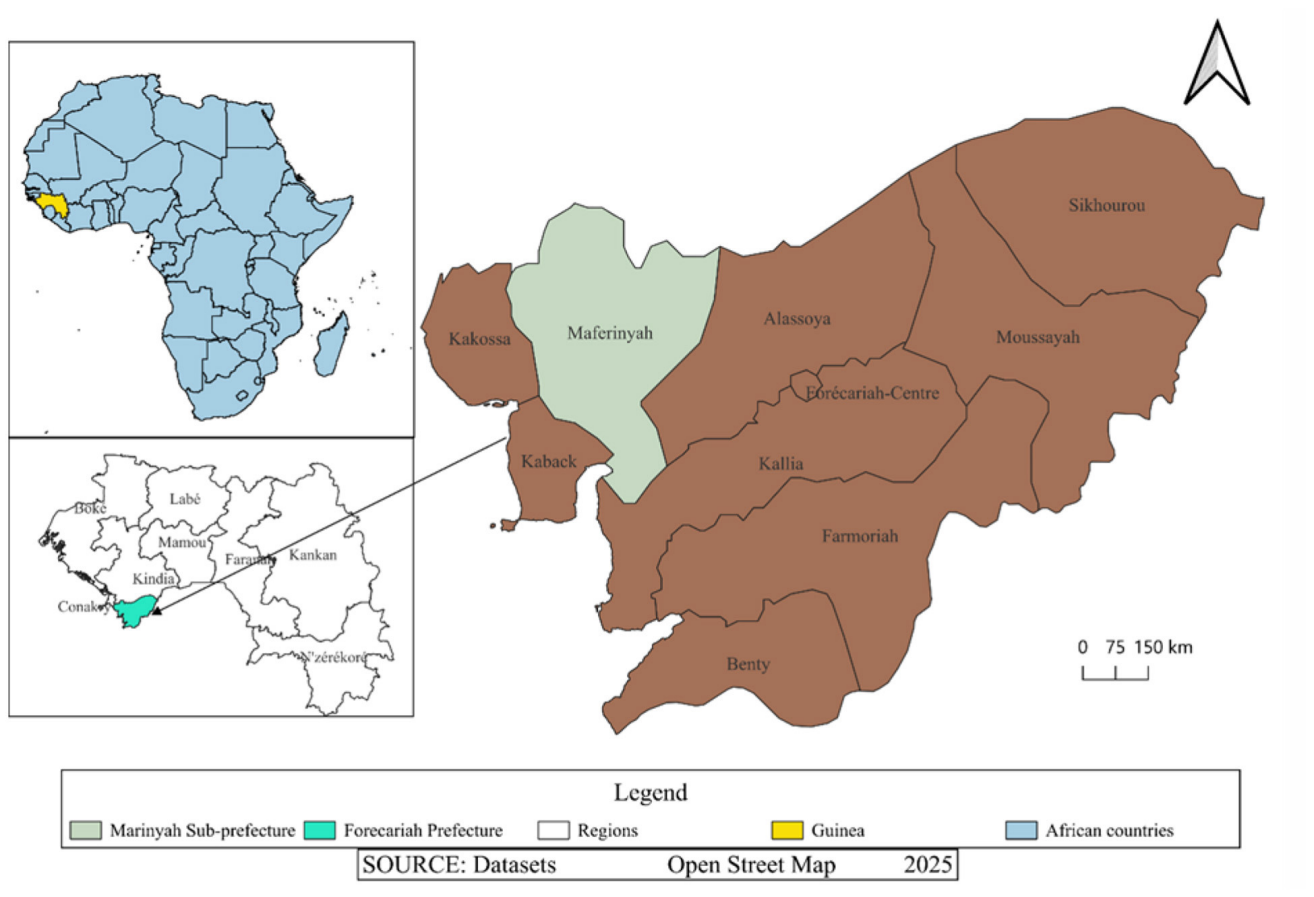
Map of the Maferinyah sub-prefecture, Guinea.

Figure 1 shows a map of the Maferinyah Sub-prefecture, Guinea. The rationale for site selection is as follows.

### 2.2 Rationale for site selection

1. Stable meso-endemic malaria burden: Maferinyah is in Forecariah Prefecture (Lower Guinea), where national surveys have reported a malaria prevalence of approximately 18% among children using microscopy in comparable ecological zones (3).
2. Intra-rural socioeconomic heterogeneity: Recent but uneven infrastructure development, including the construction of new roads, expansion of electrification, and variable access to potable water, has led to marked socioeconomic gradients within the sub-prefecture.
3. Operational feasibility: Long-standing collaborations with local health authorities and community health volunteers have facilitated rigorous monthly follow-ups, high participant retention, and the collection of reliable clinical and survey data in this rural setting.

### 2.3 Study design

**Figure 2:**
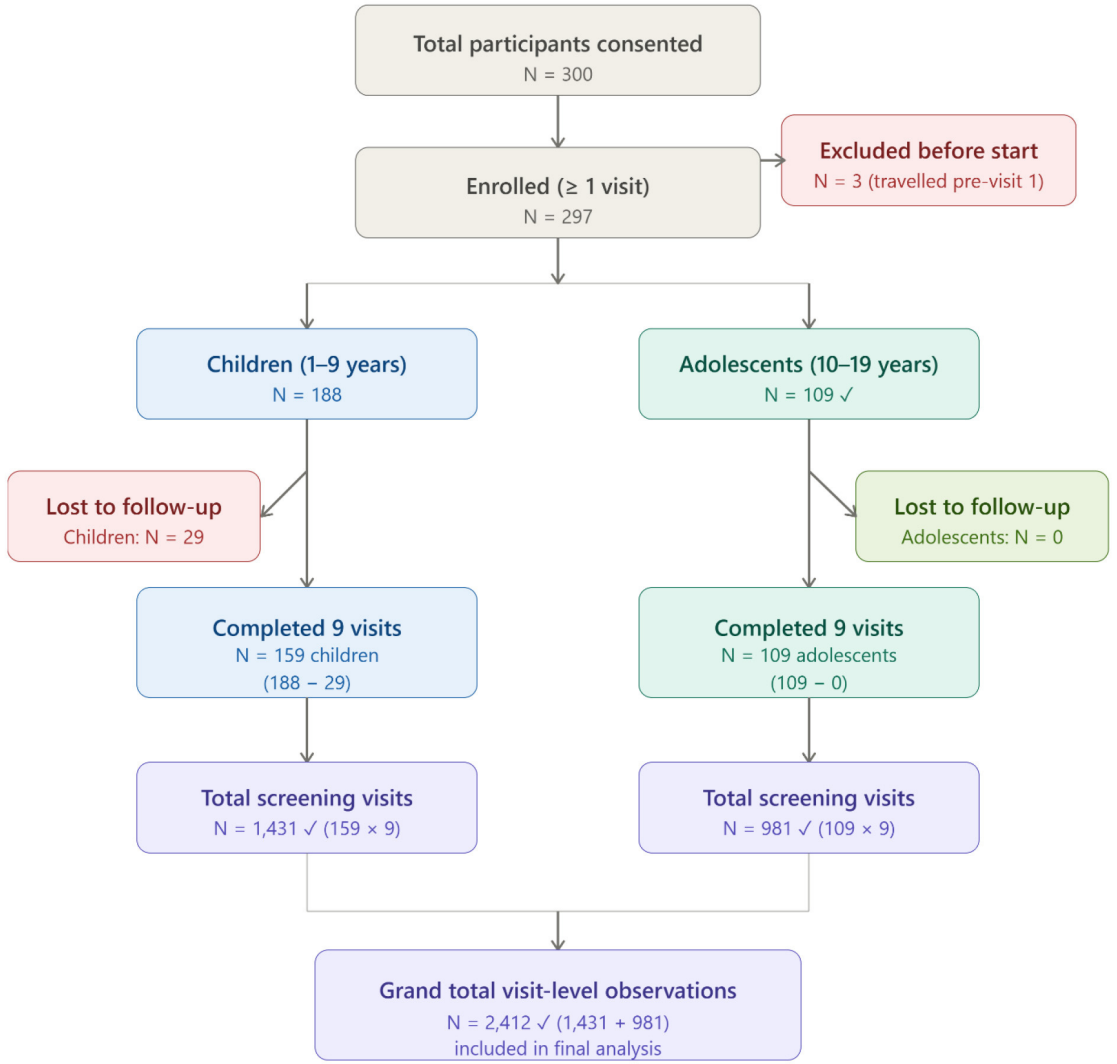
Participant flow and follow-up during a longitudinal malaria cohort study in rural Guinea.

This study employed a longitudinal design with repeated measures targeting children and adolescents living in Mafèrinyah. Data were collected through monthly home visits, with nine visits conducted between March 2022 and February 2023. Specifically, participants were followed across nine monthly or bimonthly visits scheduled at the following time points: March 2022; April–May 2022; June–July 2022; August 2022; September 2022; October–November 2022; December 2022; January 2023; and February 2023 (Figure 2), spanning a total follow-up period of 12 months and covering both the peak rainy season malaria transmission period (June–November) and the dry season low-transmission period (December–May), thereby enabling direct comparison of infection dynamics across contrasting seasonal contexts.

### 2.4 Study population and selection criteria

This study was conducted using data from a cohort previously established in the Maferinyah sub-prefecture, Guinea, as described previously (35). The cohort included children aged 1–9 years and adolescents aged 10–19 years residing in the study area. Briefly, eligible households were required to include at least three residents and at least one individual younger than 20 years. Written informed consent was obtained from the parents or legal guardians, and assent was obtained from participants aged ≥12 years. Individuals unable to adequately participate in the study procedures because of severe cognitive or psychiatric impairment or for whom participation could compromise safety or ethical protections were excluded.

### 2.5 Sampling method

At the outset of the study, a comprehensive census was conducted to identify all households in Maferinyah. This census was used to construct a complete sampling frame comprising all eligible households. Using this framework, a simple random sampling technique was applied to select the households for inclusion. This approach was chosen to ensure representativeness and minimize selection bias in the study sample.

When multiple eligible children or adolescents resided in the same household, one individual from each age group was randomly selected using a simple random method to ensure randomization and avoid selection bias. This approach aimed to balance representativeness and logistical feasibility. Importantly, even if not selected, other household members, particularly children and adolescents, had access to malaria screening and treatment during visits if they exhibited febrile symptoms, ensuring equitable treatment and health service provision across the community.

### 2.6 Sample size justification

The sample size for the parent cohort study was determined *a priori* based on expected malaria incidence estimates previously reported in similar settings and the need to ensure adequate precision for incidence estimation and age-stratified analyses, as described previously (35). Briefly, assuming an incidence rate of 0.75 malaria episodes per person-year, a 95% confidence interval, and allowance for attrition during follow-up, a total sample of approximately 300 participants was considered sufficient to support the longitudinal analyses planned for the cohort, including the multivariable modeling.

### 2.7 Study variables

#### 2.7.1 Dependent variable

The primary outcome of interest was asymptomatic malaria infection, defined as the presence of *Plasmodium* trophozoites or gametocytes in blood smears in the absence of clinical symptoms.

#### 2.7.2 Independent variables

In the present study, sociodemographic factors refer to individual- and household-level characteristics describing the social composition and positioning of participants and their households, encompassing age, sex, place of residence within the rural study area, educational level of the household head, and occupation (36). Socioeconomic status refers to the material and structural conditions of the household, including household wealth (assessed via an asset-based index), housing quality (wall, roof, and floor construction materials, and presence of open eaves), and access to and use of insecticide-treated nets (27, 37). These dimensions were chosen because they represent the most proximal structural pathways through which economic disadvantage translates into elevated vector exposure and reduced capacity for prevention and treatment, as consistently identified in the mediation literature for sub-Saharan Africa (37, 38). Together, the sociodemographic and socioeconomic dimensions examined in this study were guided by the PROGRESS framework (Place of residence, race/ethnicity/culture/language, occupation, gender/sex, religion, education, socioeconomic status, and social capital), which provides a standardized taxonomy for identifying social stratifiers relevant to health equity (35).

The explanatory variables included a range of sociodemographic and socioeconomic indicators.

- Sociodemographic factors

These included the age and sex of the participant; the study area was grouped into two analytical residential areas. Area 1 comprised Foulawa, Olympio, Maire Daouda, Tanènè, and Sarata, while Area 2 comprised Kimara, Carrefour Fodéyah, Laya, Loumafori, and Hafia, and household head characteristics, including age, gender, educational attainment, marital status, household size and occupation.

- Behavioral variables

These included access to and use of insecticide-treated nets (ITNs) and coil-based mosquito repellents (yes/no). ITN access was defined as owning an ITN within the household, whereas ITN use was defined as self-reported sleeping under an ITN the night before the survey.

- Socioeconomic indicators

Socioeconomic position (SEP) was constructed using principal component analysis (PCA) based on detailed household asset ownership and living condition variables collected at the household level. A detailed analysis of the PCA procedures, variable coding, component selection, and SEP classification is provided in Supplementary File 1. Variables included:

° Housing tenure and productive assets: home ownership, rented housing, family housing, ownership of farmland, farm ownership, and livestock ownership (e.g. cattle)
° Household assets and communication tools: ownership of a refrigerator, television, radio, telephone/mobile phone, and internet access
° Sanitation and hygiene facilities: presence of latrines, indoor toilet facilities, designated sanitation areas, and open defaecation practices.
° Cooking energy sources: use of electricity, gas, wood, or charcoal for cooking;
° Transportation and mobility assets: ownership of bicycles, motorcycles, cars, or company vehicles, as well as reliance on taxis or walking as the primary mode of transportation
° Water access and supply: primary water source (private borehole, public borehole, protected well, public tap, rainwater, or river water) and water access (within the household or compound/courtyard).

### 2.8 Data collection

Data were collected during monthly household visits using a standardized electronic questionnaire administered primarily to household heads or caregivers through the Open Data Kit (ODK) platform. Adolescents were occasionally interviewed directly when clarification or additional information was required. Follow-up visits were scheduled as follows: March 2022, April–May 2022, June–uly 2022, August 2022, September 2022, October–November 2022, December 2022, January 2023, and February 2023, thereby covering both the dry and rainy malaria transmission seasons. Data were uploaded daily to the ONA server to ensure secure storage, facilitate quality control, and manage data. The questionnaire was adapted from the established Demographic and Health Survey (DHS) instruments (34, 39).

Sociodemographic characteristics and most household socioeconomic indicators were collected at baseline, including household composition, education and occupation of the household head, housing conditions, household assets, water and sanitation characteristics, and transportation. In contrast, behavioral, clinical, and parasitological assessments were collected repeatedly during monthly follow-up visits to capture temporal and seasonal variations.

The field staff underwent 5 days of intensive training covering household interviews, temperature assessment, and biological sample collection procedures, including the preparation of thick and thin blood smears for malaria microscopy. Initial follow-up activities and participant tracing were conducted through household visits, after which participants were invited to the study research center for clinical examination, biological sample collection, and treatment when required. Monthly follow-up visits were conducted by trained clinicians who measured axillary temperature and collected information on febrile episodes occurring since the previous visit. Because participants were actively followed up and treated throughout the cohort period, symptomatic malaria episodes were relatively uncommon during follow-up. Finger-prick blood samples were collected during participant evaluations at the research center, and malaria microscopy was subsequently performed at the study laboratory by WHO-certified microscopists. Artemisinin-based combination therapy (ACT) was provided according to the national malaria treatment guidelines for participants with positive malaria test results, with referral to local health facilities when clinically indicated.

#### 2.8.1 Parasitological examinations

To ensure diagnostic accuracy and data quality, all blood smears were independently examined by two WHO-certified microscopists who were blinded to the participants' clinical information. Discrepant readings were reviewed by a third senior microscopist. In addition, 10% of the slides were randomly selected for external quality control at the National Reference Laboratory.

### 2.9 Statistical analysis

The monthly malaria prevalence was estimated as the proportion of participants with a positive parasitological result at each follow-up visit among those tested. To describe the incidence of infection over time, we calculated the monthly incidence of newly detected asymptomatic malaria infections, defined as a positive parasitological result at the current visit among participants who were negative at the preceding visit. For both indicators, proportions and corresponding 95% confidence intervals were estimated using binomial proportions based on the Wilson score method and plotted according to the month of follow-up.

For the regression analyses, asymptomatic malaria infection was analyzed at the visit level using parasitological status as a binary outcome. As participants contributed repeated observations over time, mixed-effects logistic regression models with a random intercept for participants were used to account for within-individual correlations. Univariable mixed-effects logistic regression models were first fitted to estimate the crude associations between candidate sociodemographic, socioeconomic, behavioral, and seasonal predictors and malaria infection.

A multivariable mixed-effects logistic regression model was developed using an epidemiological model-building strategy. Candidate covariates were selected *a priori* based on the literature, biological plausibility, and the study conceptual framework rather than solely on univariable statistical significance. Multicollinearity was assessed using generalized variance inflation factors derived from an equivalent fixed-effects logistic regression model, with values ≤ 3 considered acceptable. Model convergence and singularity diagnostics were also evaluated.

To investigate the potential heterogeneity in malaria risk across transmission periods, interaction terms between participant age group and season were explored. Candidate interaction models were compared with the same base model using likelihood ratio tests, Akaike information criterion (AIC), Bayesian information criterion (BIC), and singularity diagnostics. The final selected model retained the age-by-season interaction based on epidemiological plausibility and the relative model fit. Adjusted odds ratios (AORs) and corresponding 95% confidence intervals were estimated using the final mixed-effects model. The predicted probabilities and 95% confidence intervals were estimated from the final mixed-effects logistic regression model using marginal predictions with bias correction to account for the potential Jensen's inequality bias in generalized mixed-effects models.

Missing data were handled using multiple imputations by chained equations. Variables not relevant to the imputation process, including identifiers and derived variables, were excluded from the imputation and the predictor matrices. Five imputed datasets were generated, and the estimates were combined using Rubin's rules. All analyses were performed in R version 4.4.1, with statistical significance set at *p* < 0.05.

### 2.10 Ethics approval and consent to participate

Informed consent was obtained from all participants, with personal signatures or fingerprints collected to confirm voluntary participation and to allow withdrawal at any stage of the study. Informed consent was obtained from the parents or legal guardians of participants under 12 years of age. In addition, assent was obtained from all participants aged 12–19 years to ensure their understanding and voluntary participation. Community consent was obtained through discussions with village elders and family heads, followed by a comprehensive explanation of the study's objectives and procedures. Translated consent forms were provided in simple French and relevant local dialects to ensure comprehension among non-French-speaking participants. Special attention was paid to ensuring that all participants, including minors, fully understood the implications of their participation in the study. This study was conducted in accordance with the ethical standards outlined in the Declaration of Helsinki. The ethical protocol, including the consent procedures, was reviewed and approved by the National Health Ethics Committee (reference number 077/CNERS/22).

## 3 Results

### 3.1 Flowchart of inclusion

Figure 2 presents the corrected participant flow. Of 300 individuals who provided informed consent, 3 adolescents were excluded prior to study initiation as they traveled outside the study area before the first scheduled visit and did not contribute any observation. The remaining 297 participants; 188 children (aged 1–9 years) and 109 adolescents (aged 10–19 years) were enrolled and participated in at least one visit. During follow-up, 29 children were lost to follow-up after study initiation, reflecting a markedly higher retention rate in the adolescents group. A total of 268 participants completed all nine scheduled monthly visits (159 children and 109 adolescents), contributing 2,412 visit-level observations to the final analysis (1,431 children; 981 adolescents).

### 3.2 Participant characteristics at the time of recruitment

A total of 297 children and adolescents aged 1–19 years were included in the study. The participants were relatively evenly distributed across the two study areas, and the study population reflected substantial socioeconomic heterogeneity. Most households were composed of 6–12 members, and approximately half of the household heads had attained secondary education. Informal economic activities and unemployment were common, reflecting the predominantly rural socioeconomic context of the municipality. Overall, 62.0% of households reported access to at least one long-lasting insecticidal net (LLIN). Detailed sociodemographic and socioeconomic characteristics of the study population are provided in Supplementary File 2 and have been described previously in the parent cohort study (35).

### 3.3 Description of malaria prevalence

**Figure 3:**
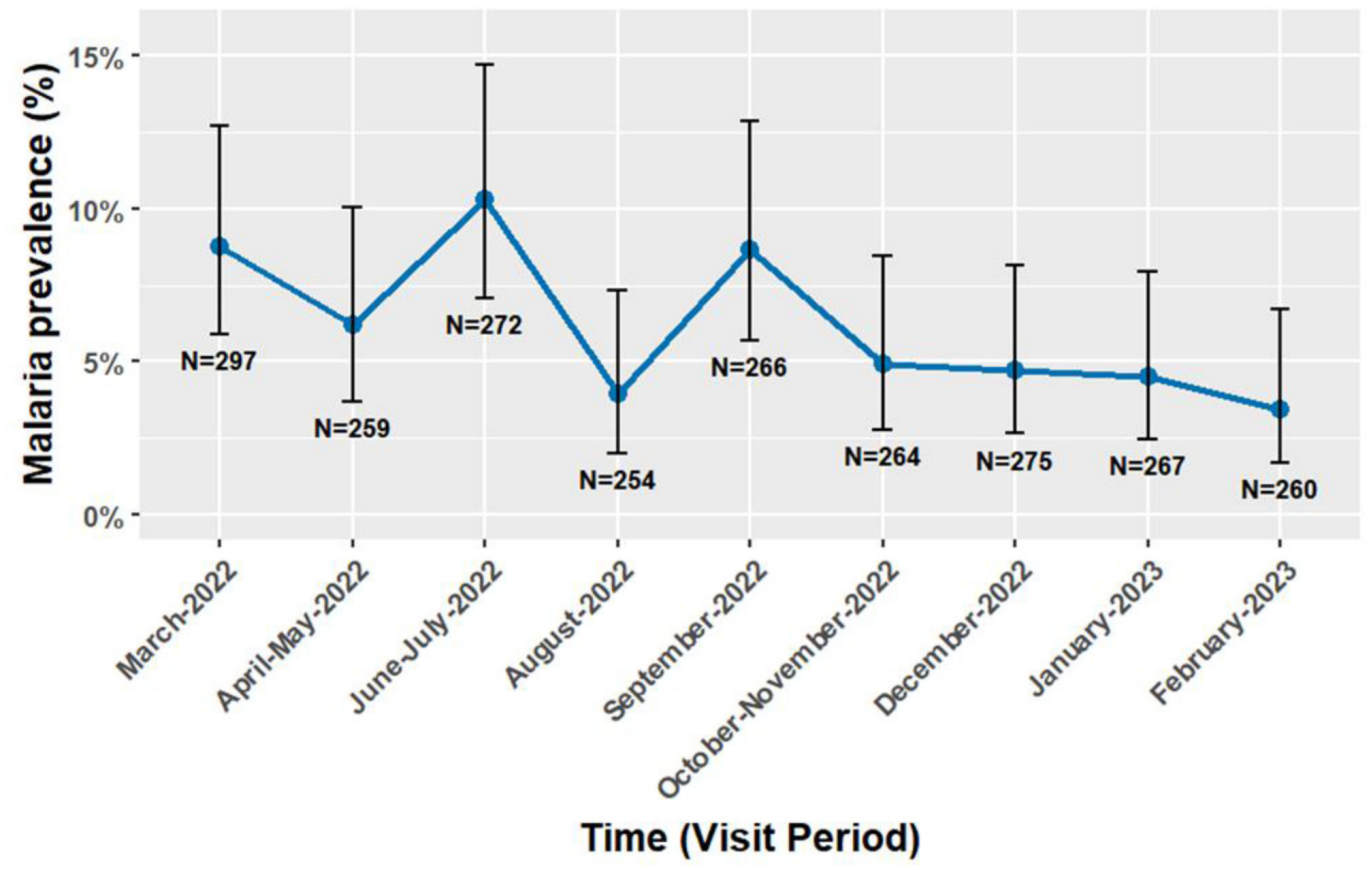
Monthly malaria prevalence during follow-up visits among children and adolescents in Mafèrinyah, Guinea, 2022–2023.

Malaria prevalence varied across monthly follow-up visits between March 2022 and February 2023 (Figure 3). The prevalence remained below 11% throughout the study period but showed noticeable temporal fluctuations. The highest prevalence was observed during June-July 2022 (10.3%, 95% CI: (7.1–14.7), followed by a secondary increase in September 2022. In contrast, the lowest prevalence was recorded in August 2022 and again toward the end of the follow-up period in February 2023. Overall, malaria prevalence tended to decline progressively from the late rainy season to the dry season. The confidence intervals overlapped across several visits, suggesting a moderate month-to-month variability.

Points represent the observed malaria prevalence at each visit; error bars indicate the 95% confidence intervals estimated using binomial methods. The sample sizes tested at each visit are displayed above the x-axis.

To examine the seasonal dimension of malaria transmission, visit-level observations were pooled into two seasons: the dry season (March 2022, December 2022, January–February 2023; four visits) and the rainy season (April–November 2022; five visits). As shown in Table 1, the prevalence of asymptomatic parasitaemia was higher during the rainy season (6.8%, 95% CI: 5.6–8.4) than during the dry season (5.5%, 95% CI: 4.2–7.0). Although this difference of 1.3 percentage points is modest in absolute terms, the confidence intervals do not overlap, suggesting a statistically meaningful seasonal contrast.

**Table 1:** Malaria prevalence by transmission season among children and adolescents in rural Guinea, March 2022–February 2023.

Pooled visit-level observations: dry season vs. rainy season				
Season	Visits pooled	N tested	Positive cases	Prevalence^*^(95% CI)
Dry season	4	1,099	60	5.5% (4.2–7.0)
Rainy season	5	1,313	90	6.8% (5.6–8.4)

^*^Dry season: March 2022, December 2022, January–February 2023 (4 visits). Rainy season: April–November 2022 (5 visits). 95% CI calculated using Wilson score method (prop.test).

### 3.4 Description of malaria incidence

**Figure 4:**
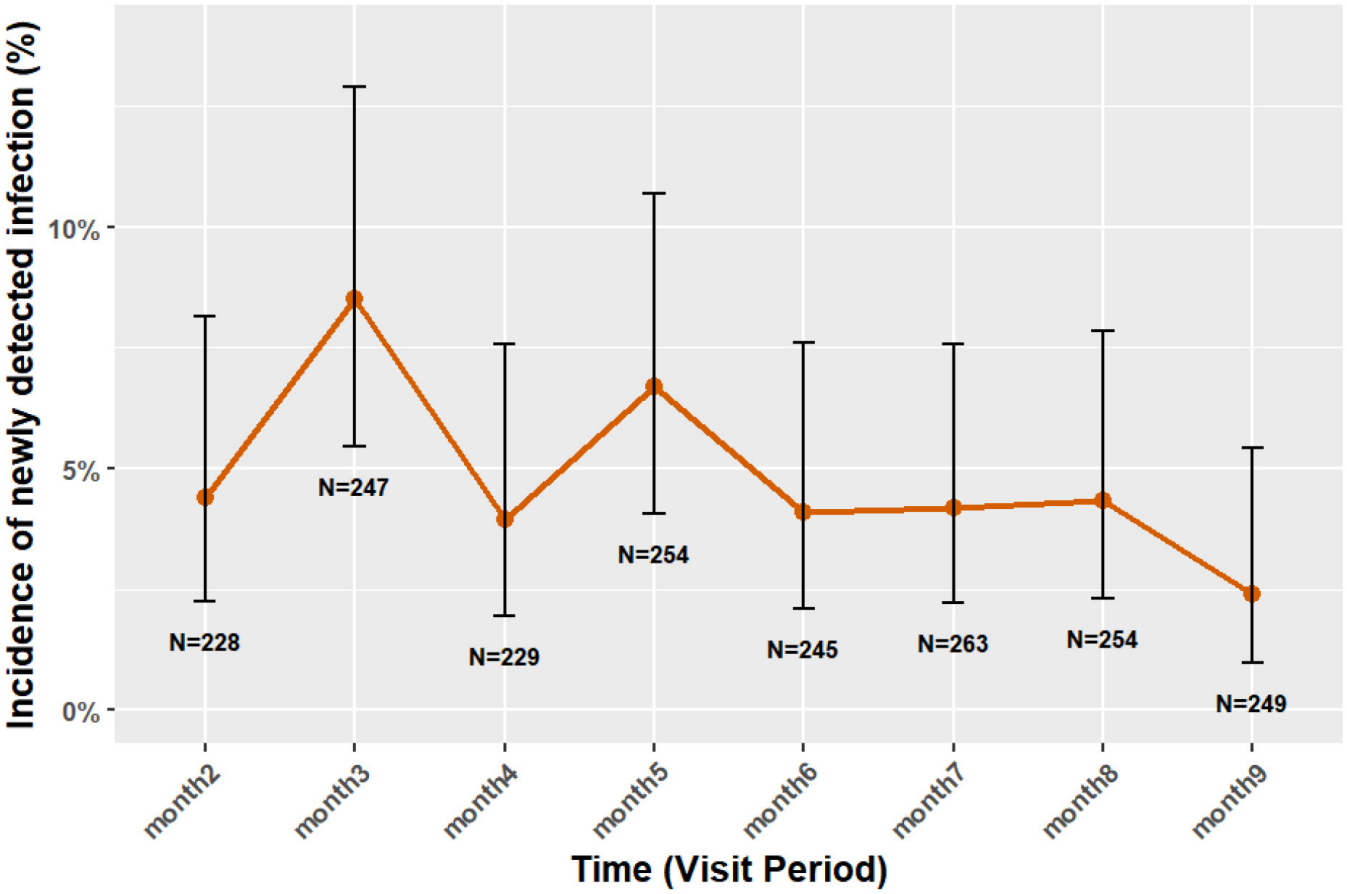
Monthly incidence of newly detected malaria infections during follow-up visits from March 2022 to February 2023.

The incidence of newly diagnosed malaria infections varied over time (Figure 4). The incidence peaked during June-July 2022, reaching 8.5% (95% CI: 5.5–12.9), consistent with the period of intensified malaria transmission during the rainy season. A smaller increase was observed in September 2022. Thereafter, the incidence gradually increased and remained relatively stable between October 2022 and January 2023 before reaching its lowest level in February 2023 (2.4%; 95% CI: 1.0–5.4). Overall, these temporal patterns indicate increased malaria transmission during the rainy season, followed by a progressive reduction during the dry season. The number of participants at risk remained relatively stable across visits, supporting the comparability of the incidence estimates over time.

Incident infection was defined as a participant who tested malaria-negative at the previous visit and malaria-positive at the current visit. Error bars represent the 95% confidence intervals estimated using binomial methods. The numbers above the x-axis indicate the number of participants at risk at each visit.

### 3.5 Univariable analysis

In univariable mixed-effects logistic regression analyses (Table 2), household head age, education, and household size were significantly associated with malaria infection. Compared with participants from households headed by individuals aged 22–34 years, those from households headed by individuals aged 35–49 years had more than threefold higher odds of malaria infection (OR: 3.24, 95% CI: 1.44–7.25), while households headed by individuals aged 50–77 years also showed increased odds (OR: 2.36, 95% CI: 1.07–5.19). Each additional household member was associated with a 77% increase in the risk of malaria infection. Secondary education of the household head was associated with lower risk of malaria infection compared with no formal education (OR: 0.51, 95% CI: 0.27–0.96).

**Table 2:** Univariable mixed-effects logistic regression of predictors of malaria infection at the visit level among children and adolescents in Mafèrinyah, Guinea, March 2022–February 2023.

Univariable mixed-effects logistic regression				
Outcome: malaria infection at visit level				
Category/unit	Negative, *n* (%)	Positive, *n* (%)	OR (95% CI)	*P*-value
Participant age category				
1–4 years	570 (94.7%)	32 (5.3%)	1.00 (Reference)	Reference
5–9 years	788 (95.2%)	40 (4.8%)	0.78 (0.38–1.61)	0.503
10–14 years	620 (92.0%)	54 (8.0%)	1.84 (0.90–3.78)	0.096
15–19 years	284 (92.2%)	24 (7.8%)	1.39 (0.56–3.44)	0.474
Household head age				
22–34 years	670 (96.4%)	25 (3.6%)	1.00 (Reference)	Reference
35–49 years	804 (92.8%)	62 (7.2%)	3.24 (1.44–7.25)	0.004
50–77 years	790 (92.6%)	63 (7.4%)	2.36 (1.07–5.19)	0.033
Household head sex				
Male	983 (94.2%)	61 (5.8%)	1.00 (Reference)	Reference
Female	1281 (93.5%)	89 (6.5%)	0.95 (0.53–1.69)	0.857
Household head education				
No formal education	689 (91.7%)	62 (8.3%)	1.00 (Reference)	Reference
Primary	321 (92.8%)	25 (7.2%)	1.16 (0.50–2.69)	0.728
Secondary	1254 (95.2%)	63 (4.8%)	0.51 (0.27–0.96)	0.038
Household head marital status				
Married	2087 (94.1%)	130 (5.9%)	1.00 (Reference)	Reference
Single	177 (89.8%)	20 (10.2%)	1.88 (0.73–4.85)	0.193
Household head occupation				
Farmer	274 (93.2%)	20 (6.8%)	1.00 (Reference)	Reference
Trader	431 (94.3%)	26 (5.7%)	0.74 (0.27–2.01)	0.549
Civil servant	338 (96.3%)	13 (3.7%)	0.54 (0.18–1.63)	0.271
Unemployed	1,221 (93.1%)	91 (6.9%)	0.91 (0.39–2.14)	0.837
ITN access				
No	821 (92.4%)	68 (7.6%)	1.00 (Reference)	Reference
Yes	1443 (94.6%)	82 (5.4%)	0.57 (0.31–1.03)	0.062
Repellent use				
No	774 (92.8%)	60 (7.2%)	1.00 (Reference)	Reference
Yes	1490 (94.3%)	90 (5.7%)	0.75 (0.43–1.30)	0.303
Socioeconomic position				
Low SEP	752 (93.6%)	51 (6.4%)	1.00 (Reference)	Reference
Middle SEP	801 (93.1%)	59 (6.9%)	0.91 (0.45–1.84)	0.786
High SEP	711 (94.7%)	40 (5.3%)	0.66 (0.31–1.41)	0.285
Participant age category				
1–4 years	570 (94.7%)	32 (5.3%)	1.00 (Reference)	Reference
5–9 years	790 (95.2%)	40 (4.8%)	0.78 (0.38–1.61)	0.503
10–14 years	620 (92.0%)	54 (8.0%)	1.84 (0.90–3.78)	0.096
15–19 years	284 (92.2%)	24 (7.8%)	1.39 (0.56–3.44)	0.474
Season				
Dry season	1282 (94.4%)	76 (5.6%)	1.00 (Reference)	Reference
Rainy season	982 (93.0%)	74 (7.0%)	1.26 (0.87–1.82)	0.215
Household size				
Per additional household member	–	–	1.77 (1.09–2.89)	0.022

Each row represents a visit-level observation. The models included a random intercept for the subject.

Table 3 shows the results of the multivariable mixed-effects logistic regression model, which included an age-by-season interaction. After adjustment, participants from households headed by individuals aged 35–49 years had significantly higher odds of malaria infection than those from households headed by individuals aged 22–34 years (aOR: 2.39, 95% CI: 1.04–5.48). Age-by-season associations were observed, particularly among participants aged 10–14 years and 15–19 years, although these associations remained statistically non-significant. No significant associations were observed for household head sex, marital status, ITN access, repellent use, socioeconomic position, or season after adjusting for confounding factors.

**Table 3:** Final mixed-effects logistic regression model for AMI, including age-by-season interaction, among children and adolescents in Mafèrinyah, Guinea, March 2022–February 2023.

Final mixed-effects logistic regression model (*N* = 2,414 visit-level observations)		
Malaria infection with age-by-season interaction		
Category	Adjusted OR (95% CI)	*P*-value
Head age		
22–34 years	–	–
35–49 years	2.39 (1.04–5.48)	0.039
50–77 years	2.39 (0.87–6.59)	0.091
Head gender		
Male	–	–
Female	1.02 (0.47–2.24)	0.957
Head education		
No formal education	–	–
Primary	1.22 (0.48–3.11)	0.676
Secondary	0.50 (0.25–1.02)	0.058
Marital status		
Married	–	–
Single	2.45 (0.84–7.16)	0.103
ITN access		
No	–	–
Yes	0.58 (0.30–1.14)	0.117
Repellent use		
No	–	–
Yes	0.75 (0.41–1.34)	0.329
Household size		
Per additional member	1.65 (0.96–2.84)	0.069
Socioeconomic position		
Low SEP	–	–
Middle SEP	1.54 (0.70–3.37)	0.284
High SEP	1.02 (0.43–2.44)	0.962
Age group		
1–4 years	–	–
5–9 years	0.99 (0.42–2.35)	0.979
10–14 years	0.92 (0.36–2.37)	0.870
15–19 years	2.42 (0.87–6.68)	0.089
Season		
Dry season	–	–
Rainy season	1.35 (0.61–2.97)	0.461
Age × season interaction		
1–4 × dry	–	–
5–9 × rainy	0.54 (0.18–1.59)	0.266
10–14 × rainy	2.44 (0.86–6.92)	0.094
15–19 × rainy	0.27 (0.07–1.02)	0.054

Reference categories are shown as (–). The model included a random intercept for Subject ID.

### 3.6 Predicted probabilities of malaria infection in children and adolescents from March 2022 to February 2023

**Figure 5:**
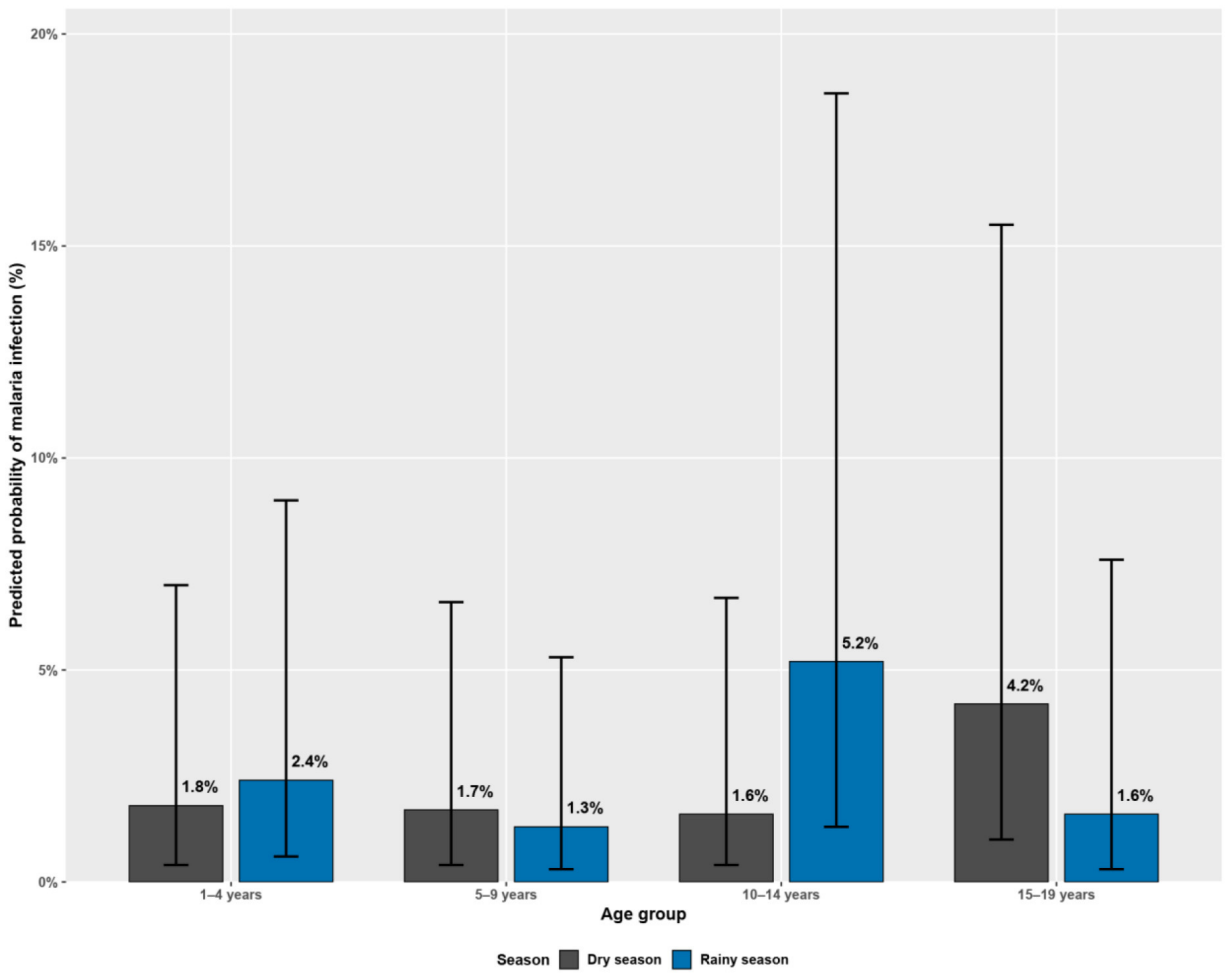
Predicted probability of malaria infection by age group and season among children and adolescents in Maferinyah, Guinea, 2022–2023.

The predicted probabilities from the mixed-effects logistic regression model showed substantial variation in malaria infection risk across age groups and during different seasons (Figure 5). During the rainy season, adolescents aged 10–14 years exhibited the highest predicted probability of malaria infection (5.2%, 95% CI: 1.3–18.6), compared with the lower probabilities observed among younger children aged 1–4 years (2.4%, 95% CI: 0.6–9.0) and 5–9 years (1.3%, 95% CI: 0.3–5.3). In contrast, during the dry season, the highest predicted probability was observed among adolescents aged 15–19 years (4.2%, 95% CI: 1.0–15.5).

Predicted probabilities were estimated from the final mixed-effects logistic regression, including an interaction between age group and season and a random intercept for participants. Error bars represent 95% confidence intervals derived from model-based standard errors on the logit scale and transformed back to the probability scale using the inverse logit function. The bars represent the adjusted predicted probabilities of malaria infection for each age group-season combination. Wider confidence intervals observed for some subgroups likely reflect limited precision due to the relatively small number of observations within specific age–season combinations.

## 4 Discussion

### 4.1 Household socioeconomic and social determinants of malaria infection

This longitudinal cohort study provides novel evidence of household-level socioeconomic and contextual determinants of asymptomatic malaria infection among children and adolescents in rural Guinea. Using repeated monthly observations, we identified significant associations between malaria infection and household head age, educational attainment, and marital status, highlighting the heterogeneity in malaria risk across age groups and transmission seasons. These findings suggest that malaria vulnerability in this setting is shaped not only by environmental exposure but also by broader household sociodemographic and socioeconomic characteristics of the population.

One of the most notable findings was the higher odds of malaria infection among children and adolescents living in households headed by individuals aged 35–49 years compared to those headed by younger adults aged 22–34 years. In our cohort, household heads aged 35–49 years also had the highest proportion of no formal education, which may partly explain the observed increase in malaria odds. Lower educational attainment can influence malaria prevention through reduced access to health information, lower uptake of preventive behaviors, and weaker engagement with health services and vector control interventions. Similar pathways linking educational disadvantages and malaria risk have been documented across sub-Saharan Africa (40). A recent systematic review reported that higher educational attainment was consistently associated with a lower malaria risk through improved prevention practices, housing conditions, and healthcare utilization (20). However, the association between household head age and malaria infection remained elevated after adjusting for educational level in the multivariable analysis, suggesting that additional social and contextual mechanisms may also contribute to this relationship, and that the observed association is unlikely to be explained by educational disparities alone and may additionally reflect occupational and caregiving constraints operating at the household level.

In this context, the observed association may further reflect the socioeconomic and occupational realities of the Maferinyah rural community. Adults aged 35–49 years represent the most economically active population group, commonly engaged in farming, trading, and other income-generating activities that require substantial daily mobility and time commitment. Because the cohort involved active monthly follow-up visits and free malaria care requiring caregivers to bring participants to the health center, competing occupational demands may have reduced the regular availability of some household heads for timely preventive engagement and follow-up participation (38). In contrast, younger and older household heads may have had greater flexibility or availability to accompany participants to scheduled visits. Although all participants received active surveillance and free treatment, differential availability for routine follow-up may have indirectly influenced malaria detection, treatment timing and adherence to preventive recommendations throughout the study period.

### 4.2 Age-specific seasonal patterns and behavioral exposure

The predicted probabilities of malaria infection varied markedly across age groups and seasons; the highest probability was observed among adolescents aged 10–14 years during the rainy season and among those aged 15–19 years during the dry season. These contrasting age-season profiles are epidemiologically important and suggest that distinct mechanisms drive the infection risk in younger vs. older adolescents, depending on the transmission context. The higher predicted probability among 10–14-year-olds during the rainy season is epidemiologically plausible because this age group corresponds to school-aged children, who are increasingly recognized as an important reservoir of malaria infection in sub-Saharan Africa (6, 8, 41–43). They often experience frequent asymptomatic or low-symptom infections and may be less consistently targeted by malaria control strategies than children aged under 5 years. During the rainy season, increased vector density and outdoor exposure related to school, play, and household activities may amplify this risk. In many rural settings, children and young adolescents commonly participate in evening household activities, such as cooking preparation, cleaning, water collection, and other outdoor domestic tasks that occur during peak Anopheles mosquito biting periods. In addition, social interaction and playing activities in the evening may increase exposure before bedtime and ITN use. Previous studies have shown that outdoor exposure and behavioral routines among school-aged children can contribute substantially to residual malaria transmission, despite household-level vector control interventions (44, 45).

In contrast, the higher predicted probability among 15–19-year-olds during the dry season may suggest a different mechanism. A modeling analysis using longitudinal cohort data from Mali, a comparable West African seasonal transmission setting, demonstrated that dry season carriage of Plasmodium *falciparum* is not purely stochastic but is disproportionately concentrated in the oldest, most exposed, and most immune individuals within a population, who are predicted to enable a more than twofold faster re-sending of infection into the mosquito population at the onset of the subsequent wet season (46). This exposure-dependent carriage pattern provides a mechanistic basis for understanding why older adolescents in our study may retain detectable parasitaemia during low-transmission months: having accumulated greater cumulative exposure and partial immunity over successive transmission seasons, 15–19 years-olds are more likely than younger children to sustain low-density infections through the dry season without progressing to clinical disease, the hallmark of asymptomatic parasite carriage (46). This interpretation extends the findings of Stadler et al. beyond their original pediatric cohort (46) and is consistent with broader evidence that older children and adolescents act as an important inter-seasonal reservoir for Plasmodium *falciparum* infections (47). However, persistent carriage alone may not fully explain the observed patterns. Older adolescents may also experience continued low-level exposure during the dry season through evening social activities, occupational tasks, or outdoor mobility patterns that increase contact with residual mosquito populations, despite lower overall vector abundance. In rural settings, adolescents aged 15–19 years often have greater autonomy and mobility than younger children, potentially extending outdoor exposure into evening hours when mosquito bites may still occur. Residual malaria transmission during the dry season has been linked to behavioral exposure outside the periods of optimal bed net protection (44, 48). These findings support the growing recognition that residual malaria transmission may persist outside peak transmission periods through behavioral exposure patterns that are insufficiently addressed by conventional indoor-focused interventions.

### 4.3 Public health implications, limitations, and future directions

The findings of this study have important implications for malaria control strategies targeting school-aged children and adolescents in rural endemic settings. Current malaria interventions in many sub-Saharan African countries remain strongly focused on children aged under 5 years and pregnant women, potentially overlooking older children and adolescents, who may contribute substantially to persistent asymptomatic parasite reservoirs. The observed heterogeneity in malaria risk according to household social structure and seasonal behavior suggests that malaria prevention strategies may benefit from greater integration of household-level socioeconomic realities and adolescent-specific exposure patterns.

This study has several limitations. First, the primary outcome, asymptomatic malaria infection, was measured exclusively using light microscopy, which, while widely used and field-adaptable, has limited sensitivity for detecting low-density parasitaemia, particularly in asymptomatic patients. More sensitive diagnostic methods, such as polymerase chain reaction (PCR) or loop-mediated isothermal amplification (LAMP), would enhance detection accuracy, especially for submicroscopic infections. Consequently, our findings may underestimate the true burden of asymptomatic malaria, and misclassification bias could have attenuated some observed associations. Second, the duration of the study was limited to an eleven-month period, which restricts the ability to assess full year-round transmission patterns or multi-year trends. Therefore, caution is warranted when generalizing these findings to broader seasonal contexts. Third, although we examined a broad array of sociodemographic and socioeconomic factors, the study did not exhaustively capture all potential determinants of malaria risk, such as environmental exposures (e.g., proximity to breeding sites), access to healthcare services, or social capital. These unmeasured confounders may also shape malaria risk, underscoring the need for more integrative research in the future. Lastly, while clinical indicators such as parasite density and hemoglobin levels are biologically meaningful in malaria research, we intentionally excluded them from multivariable models to avoid over-adjustment and to retain a clear analytical focus on upstream social determinants. Future studies could strengthen this approach by employing causal mediation frameworks that jointly model clinical severity and social risk factors.

Despite these limitations, this study provides foundational evidence of the social and structural drivers of malaria in a rural West African context. The insights offered here may serve as a critical platform for developing more targeted, equitable, and context-sensitive malaria control strategies.

Future research should further explore the behavioral, occupational, and social mechanisms underlying age- and household-related differences in malaria risk, including the role of caregiving dynamics, mobility, and access to prevention among vulnerable households. Longitudinal studies incorporating behavioral and spatial data could improve the understanding of persistent malaria transmission among older children and adolescents in endemic rural settings.

## 5 Conclusion

These findings highlight the need for malaria control strategies in rural Guinea that move beyond universal approaches and incorporate age-sensitive and socially responsive interventions. Older children and adolescents should receive greater attention within malaria surveillance and prevention programs, particularly during seasonal transmission peaks, given their potential contribution to sustaining asymptomatic parasite reservoirs within the community. The study also underscores the importance of addressing broader household and social vulnerabilities that may influence engagement in malaria prevention and follow-up activities. Community-based strategies adapted to the realities of economically active households, including flexible outreach approaches and strengthened health education targeting caregivers, may improve the continuity of preventive care and early detection of infection.

## Data Availability

The original contributions presented in the study are included in the article/Supplementary material, further inquiries can be directed to the corresponding author.
