# Supplementary material for: Sociodemographic and socioeconomic predictors of asymptomatic malaria among children and adolescents in rural Guinea: a longitudinal study
Source: Front Public Health. 2026 Jul 23;14:1891980. doi: 10.3389/fpubh.2026.1891980 (PMC13442431; doi:10.3389/fpubh.2026.1891980)
Supplement: Supplementary file 1 [file Supplementary_file_1.docx]

**Sociodemographic and Socioeconomic predictors of asymptomatic malaria among children and adolescents in rural Guinea: A longitudinal study.**

|  | **[ALL]** | **N** |
| --- | --- | --- |
| **Characteristics** | **N=297** |  |
| Residence |  | 297 |
| area1 | 45.1% [39.4%;51.0%] |  |
| area2 | 54.9% [49.0%;60.6%] |  |
| Head of household education |  | 297 |
| No formal education | 32.3% [27.0%;38.0%] |  |
| Primary | 15.8% [11.9%;20.5%] |  |
| Secondary | 51.9% [46.0%;57.7%] |  |
| Head of household gender |  | 297 |
| Men | 43.8% [38.0%;49.6%] |  |
| Women | 56.2% [50.4%;62.0%] |  |
| Head of household marital status |  | 297 |
| Married | 91.6% [87.8%;94.5%] |  |
| Single | 8.42% [5.52%;12.2%] |  |
| Head of household occupation |  | 297 |
| Farmer | 13.8% [10.1%;18.3%] |  |
| Merchant | 19.2% [14.9%;24.1%] |  |
| Civil servant | 13.5% [9.80%;17.9%] |  |
| Unemployed | 53.5% [47.7%;59.3%] |  |
| Number of household members |  | 297 |
| [3,5] | 21.9% [17.3%;27.0%] |  |
| [6,12] | 61.3% [55.5%;66.9%] |  |
| [13,35] | 16.8% [12.8%;21.6%] |  |
| Use of mosquito repellent |  | 297 |
| No | 35.7% [30.2%;41.4%] |  |
| Yes | 64.3% [58.6%;69.8%] |  |
| Wealth quintile |  | 297 |
| Low socioeconomic position (SEP) | 35.4% [29.9%;41.1%] |  |
| Middle_SEP | 34.3% [29.0%;40.0%] |  |
| High SEP | 30.3% [25.1%;35.9%] |  |
| Insecticide Treated Net access |  | 297 |
| No | 38.0% [32.5%;43.8%] |  |
| Yes | 62.0% [56.2%;67.5%] |  |
| Participants group age |  | 297 |
| [1,4] | 26.6% [21.7%;32.0%] |  |
| (4,9] | 36.7% [31.2%;42.5%] |  |
| (9,14] | 23.9% [19.2%;29.2%] |  |
| (14,19] | 12.8% [9.22%;17.1%] |  |

```
prop.table((table(dataeduc$Head_age,dataeduc$Head_education)),2)

          no_formal_education    primary  secondary
  [22,35)          0.19791667 0.55319149 0.24675325
  [35,50)          0.42708333 0.38297872 0.29870130
  [50,77]          0.37500000 0.06382979 0.45454545
```
