# Supplementary material for: Sociodemographic and socioeconomic predictors of asymptomatic malaria among children and adolescents in rural Guinea: a longitudinal study
Source: Front Public Health. 2026 Jul 23;14:1891980. doi: 10.3389/fpubh.2026.1891980 (PMC13442431; doi:10.3389/fpubh.2026.1891980)
Supplement: Supplementary file 2 [file Supplementary_file_3.docx]

**Sociodemographic and Socioeconomic predictors of asymptomatic malaria among children and adolescents in rural Guinea: A longitudinal study.**

**Construction of the socioeconomic position (SEP) index using principal component analysis (PCA).**

**Conceptual justification for using PCA:**

Socioeconomic position (SEP) was assessed using an asset-based wealth index derived from principal component analysis (PCA). In rural and peri-rural settings in sub-Saharan Africa, direct measures of household income or expenditure are often difficult to obtain reliably because income is frequently seasonal, informal, and unstable. Consequently, household asset ownership and housing characteristics are widely used as proxy indicators of long-term socioeconomic conditions in demographic and health studies.

PCA is a data-reduction technique that identifies the combination of variables explaining the greatest variation across households and has been extensively used in Demographic and Health Surveys (DHS), malaria indicator surveys (MIS), and equity analyses to construct relative wealth indices. The first principal component is generally interpreted as representing the underlying socioeconomic gradient in the study population.

Variables included in the PCA

All variables were binary indicators coded as follows:

- 0=No/absence
- 1=Yes/ presence

The variables included in the PCA are as follows:

| **Domain** | **Variable description** |
| --- | --- |
| Housing tenure | Owns the house |
| Housing tenure | Lives in rented housing |
| Housing tenure | Lives in a family house |
| Productive assets | Owns a farm |
| Productive assets | Owns livestock/cattle |
| Productive assets | Owns agricultural land |
| Household assets | Owns a refrigerator |
| Household assets | Owns a television |
| Household assets | Owns a radio |
| Household assets | Owns a telephone/mobile phone |
| Household assets | Has internet access |
| Sanitation | Has a latrine |
| Sanitation | Practices open defecation |
| Sanitation | Has a toilet inside the house |
| Sanitation | Has an arranged sanitation area |
| Cooking energy | Uses electricity for cooking |
| Cooking energy | Uses gas for cooking |
| Cooking energy | Uses wood for cooking |
| Cooking energy | Uses charcoal for cooking |
| Housing materials | Cement wall |
| Housing materials | Baked-earth wall |
| Housing materials | Clay wall |
| Housing materials | Wooden wall |
| Roofing materials | Metal-sheet roof |
| Roofing materials | Tile roof |
| Roofing materials | Thatched roof |
| Transport assets | Owns a bicycle |
| Transport assets | Owns a motorcycle |
| Transport assets | Owns a car |
| Transport assets | Owns a company vehicle |
| Transport assets | Use taxi transportation |
| Transport assets | No transport means/walking |
| Water source | Borehole water |
| Water source | Closed well |
| Water source | Rainwater |
| Water source | River water |
| Water source | Water source inside the house |
| Water source | Water source inside the compound |
| Water source | Public well |
| Water source | Public borehole |
| Water source | Public tap |

**Coding procedure**

All variables were transformed into numerical indicators before the PCA estimation.

**Pre-processing steps**

Prior to PCA estimation, the following preprocessing procedures were applied.

Missing values for asset variables were recorded as 0 (absence of an asset or characteristic).

Participant identifiers were removed from the PCA datasets.

Variables with zero variance were excluded because they did not contribute to the discrimination between households.

All variables were standardised before the PCA using z-score transformation to ensure comparability across variables.

After preprocessing, two variables showed no variability and were excluded.

- Tile roof
- Company vehicle ownership

**PCA estimation procedure**

PCA was performed on the standardised correlation matrix of socioeconomic variables using the prcomp() function in R, with scaling enabled.

The PCA generated orthogonal components representing independent dimensions of variation in the socioeconomic characteristics of households.

The eigenvalues and explained variance indicated that the first principal component (PC1) captured the dominant socioeconomic gradient.

| **Component** | **Eigenvalue** | **Variance explained (%)** | **Cumulative variance (%)** |
| --- | --- | --- | --- |
| PC1 | 13.14 | 32.1 | 32.1 |
| PC2 | 3.19 | 7.8 | 39.8 |
| PC3 | 2.37 | 5.8 | 45.6 |

**Component selection**

The first principal component (PC1) was retained as the SEP index for the following reasons:

- This explained the largest proportion of the total variance.
- This represents the principal socioeconomic gradient across households.
- This approach is consistent with the standard DHS and malaria equity methodologies.
- Positive factor loadings on PC1 were generally associated with ownership of household assets and improved housing conditions, indicating a higher socioeconomic position.

Examples of variables with relatively high positive loadings included:

- Mobile phone ownership
- Television ownership
- Refrigerator ownership
- Improved sanitation indicators
- Improved housing materials

**Construction of the wealth score**

The SEP score for each household was derived from the first principal component using the following weighted linear combination:

where:

- $SEP_{i}$= socioeconomic score for household $i$
- $X_{ik}$= standardized value of asset variable $k$
- $w_{k}$= loading (weight) of variable $k$on the first principal component
- $p$= number of included variables

Higher scores indicate a higher relative socioeconomic position.

The distribution of the first principal component showed a clear separation across wealth groups.

| SEP group | Mean PC1 score |
| --- | --- |
| Low SEP | −4.11 |
| Middle SEP | 1.12 |
| High SEP | 3.00 |

**Categorisation into SEP groups**

The continuous PC1 wealth score was ranked and divided into tertiles using equally sized groups. Households were subsequently categorised as follows:

- Low SEP
- Middle SEP
- High SEP

The resulting groups were balanced in terms of size.

| SEP category | Number of observations |
| --- | --- |
| Low SEP | 1,071 |
| Middle SEP | 1,070 |
| High SEP | 1,070 |

These SEP categories were used in all subsequent analyses to examine socioeconomic inequalities in malaria prevention indicators, including LLIN access, LLIN use, and malaria transmission outcomes.
